# *Fv1* Restriction and Retrovirus Vaccine Immunity in *Apobec3*-Deficient 129P2 Mice

**DOI:** 10.1371/journal.pone.0060500

**Published:** 2013-03-22

**Authors:** Kalani Halemano, Bradley S. Barrett, Sam X. Li, Michael S. Harper, Diana S. Smith, Karl J. Heilman, Mario L. Santiago

**Affiliations:** 1 Department of Medicine, University of Colorado Denver, Aurora, Colorado, United States of America; 2 Department of Microbiology, University of Colorado Denver, Aurora, Colorado, United States of America; 3 Integrated Department of Immunology, University of Colorado Denver, Aurora, Colorado, United States of America; National Institute of Allergy and Infectious Diseases, United States Of America

## Abstract

Understanding the host genetics of the immune response in retrovirus infection models could provide insights for basic HIV vaccine discovery. In Friend retrovirus (FV) infection of mice, *Fv1* differentially inhibits N-tropic versus B-tropic FV infection by mediating a capsid-dependent post-entry block, *Fv2* susceptibility governs splenomegaly induction, and *Rfv3* resistance primes a stronger neutralizing antibody response due to more potent Apobec3 activity. *Apobec3* polymorphisms in inbred mouse strains correlate with *Rfv3* resistance and susceptibility, with one unresolved exception. The 129/OlaHsd (129P2) mouse strain is *Fv2* and *Rfv3* susceptible based on genotyping, but infection of 129P2 mice with B-tropic FV resulted in strong neutralizing antibody responses and no splenomegaly. Here we confirm that 129P2 mice are *Fv1^nr/nr^,* explaining its resistance to B-tropic FV. Infection of 129P2 mice with NB-tropic FV, which can efficiently infect mice independent of *Fv1* genotype, resulted in severe splenomegaly, high levels of viremia and weak neutralizing antibody responses regardless of *Apobec3* status. Notably, high-dose B-tropic FV infection of 129P2 *Apobec3*-deficient mice induced significant adaptive immune responses and conferred high levels of protection following challenge with pathogenic NB-tropic FV. This immunological protection complemented previous studies that N-tropic FV can act as a live-attenuated vaccine in *Fv1* ^b/b^ mice. Altogether, the results obtained in 129P2 mice strengthen the conclusion that *Rfv3* is encoded by *Apobec3*, and highlight *Fv1* incompatibility as a retroviral vaccine paradigm in mice. Due to its susceptibility to disease that allows for pathogenic challenge studies, B-tropic FV infection of 129P2 mice may be a useful model to study the immunological pathways induced by retroviral capsid restriction.

## Introduction

The innate arm of the immune system could critically shape the adaptive immune response against pathogens. Major efforts to understand these innate immune mechanisms against HIV-1 resulted in the identification of restriction factors such as TRIM5α [1] and APOBEC3G [2], but how these factors shape adaptive immune responses against HIV-1 is difficult to study in humans *in vivo*. In contrast, the interplay between innate and adaptive immunity has been studied extensively in the Friend retrovirus (FV) infection model [3]–[5]. FV is a complex of a replication-competent Friend Murine Leukemia Virus (F-MuLV) and a replication-defective Spleen Focus Forming Virus (SFFV), that cause severe splenomegaly and erythroleukemia in mice [3]–[5]. Different inbred mouse strains exhibited distinct resistance and susceptibility to FV infection and disease that mapped to a handful of genes that include *Fv1, Fv2,* and *Rfv3* [3]–[5] (Table 1). Interestingly, *Fv1* and *Rfv3* are the functional counterparts of human *TRIM5α* and *APOBEC3G*, respectively [6]–[8]. Thus, understanding the impact of *Fv1* and mouse *Apobec3* (or *mA3*) on FV adaptive immunity may provide insights for basic HIV vaccine discovery.

**Table 1:** FV genotype status of various inbred mouse strains.

	Genotype^a^			
Host strain	*Fv1*	*Fv2*	*Rfv3*	References
C57BL/6 (B6)	*b/b*	*r/r*	*r/r*	[7], [12], [13]
C57BL/10 (B10)	*b/b*	*r/r*	*r/r*	[13]
BALB/c	*b/b*	*s/s*	*s/s*	[7], [12], [13]
A.BY^b^	*b/b*	*s/s*	*s/s*	[7], [12]
A/WySn^b^	*b/b*	*s/s*	*s/s*	[11], [12], [13]
129/OlaHsd (129P2)	*nr/nr*	*s/s*	*s/s*	This study

a *Fv1* controls capsid-dependent tropism; *Fv2* is a dominant susceptibility (*s*) gene governing splenomegaly; *Rfv3* resistance *(r*) is associated with a stronger NAb response. ^b^A/WySn mice also have a defective B-cell activating receptor gene, *BAFF-R*, that maps near the *Rfv3* locus [11], [12], but this was not observed in the related A.BY mice [12].

*Rfv3* is a classical gene that influences recovery from FV viremia by promoting a strong neutralizing antibody (NAb) response [9], [10]. Its molecular identification as *Apobec3* [7], [11], [12] raised intriguing implications for HIV-1 immunity, since the human homologue APOBEC3G is counteracted by the HIV-1 protein Vif [2]. The case for *Apobec3* as the gene encoded by *Rfv3* was primarily built on evidence from F_1_ transcomplementation studies [7], [11], [12]. However, this evidence was also supported by the strong correlation between *Apobec3* polymorphisms and the *Rfv3* genotype of the inbred strains used to identify and map the *Rfv3* gene. Compared to the *Apobec3* alleles of *Rfv3* susceptible mice such as BALB/c, A/WySn and A.BY strains, *Rfv3* resistant C57BL mice (B6 or B10) mice exhibit: (1) high *Apobec3* mRNA expression levels [11]–[16] that was linked to a 530 bp Xenotropic Murine Leukemia Virus Long Terminal Repeat (X-MLV) insertion at the *Apobec3* exon 2 splice site [12], [16]; (2) splicing of *Apobec3* exon 5, resulting in increased translation of an isoform with more potent antiretroviral activity [13], [17]–[19]; and (3) amino acid changes potentially flanking the putative polynucleotide-accommodating groove [16]. These differences could all account for why the *Rfv3* resistant allele of *Apobec3* is more potent at restricting FV than the *Rfv3* susceptible allele *in vivo*. However, it remains unknown whether the *Rfv3* susceptible *Apobec3* allele could promote recovery from FV viremia and NAb responses compared to *Apobec3*-deficient mice.

**Figure 1:**
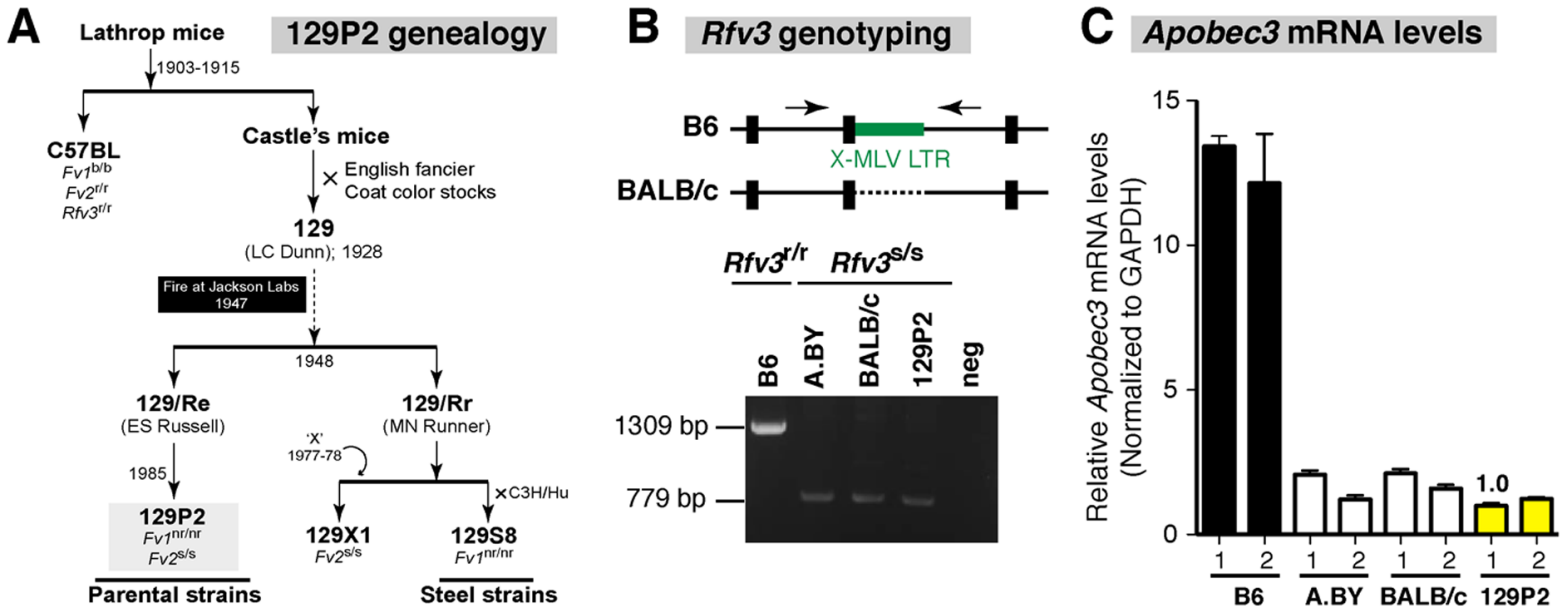
Resolving the *Rfv3* status of the 129P2 strain. (A) Genealogy of 129P2 mice. Two sublines, 129/Re and 129/Rr, gave rise to contemporary Parental and Steel strains, respectively. 129X1 was genetically contaminated by an unknown strain ‘X’ [21], [22], and genotyped later as *Fv2* ^s/s^ [24]. The Steel substrain 129S8 was previously genotyped as *Fv1* ^nr/nr^ [33], [36]. As expected, 129P2 mice had the same *Fv1* and *Fv2* genotype, suggesting that the entire 129 lineage are *Fv1* ^nr/nr^ and *Fv2* ^s/s^. (B) *Rfv3* genotyping. Primers flanking a 530-bp X-MLV LTR insertion in B6 *Apobec3* were used. PCR products visualized by agarose electrophoresis revealed that 129P2 mice lacked the insert and are therefore *Rfv3* susceptible. (C) *Apobec3* mRNA levels. Spleen mRNA were extracted and subjected to quantitative PCR using *Apobec3-*specific primers and normalized to GAPDH levels [12]. Relative to B6 mice, 129P2 mice had >10-fold lower *Apobec3* mRNA levels. Two mice from each strain were tested. Error bars correspond to standard deviations from triplicate determinations.

The *Apobec3* polymorphisms in the inbred mouse strains used to define *Rfv3* are highly concordant. However, the *Apobec3/Rfv3* status of a more recently studied mouse strain in the FV infection model, 129/OlaHsd (129P2 [20]; cited previously as 129/Ola [7]), remains unclear. 129P2 is a substrain of a diverse family of inbred mice under the generic 129 background ([20]–[22]; Fig. 1A), and was used extensively for gene-targeting studies, including *Apobec3* [7]. In a previous study, 129P2 mice were classified as *Rfv3* resistant because high-dose infection with B-tropic FV resulted in undetectable viremia and potent NAb responses by 28 days post-infection (dpi), similar to B6 mice [7]. However, quantitative PCR data showing that 129P2 mice had relatively high *Apobec3* mRNA levels were incorrect [12]. The *Fv2* genotype of 129P2 mice also needed to be clarified. *Fv2* is a dominant susceptibility gene that dictates splenomegaly induction and is encoded by the *Stk* gene [23], [24]. In *Fv2* susceptible mice, a 3 nt (GGA) insertion in the *Stk* intron 10 of *Fv2* susceptible strains results in an alternative promoter that drives the transcription of a short-form of the Stk kinase (sf-Stk) [24]. Sf-Stk interacts with the erythropoietin receptor in conjunction with the SFFV gp55^P^ protein, resulting in the uncontrolled proliferation of erythroblast precursors that leads to severe splenomegaly [25], [26]. Since 129P2 mice did not develop splenomegaly following B-tropic FV infection, 129P2 mice were classified as *Fv2* resistant [7]. However, a different 129 substrain, 129X1 (formerly 129/SvJ [20]), was genotyped as *Fv2* susceptible [24]. Since 129 mice were long separated from the *Fv2* resistant C57BL lineage [20]–[22], [27], it is unlikely that 129P2 mice are *Fv2* resistant. However, due to the complex genealogy of the 129 lineage (Fig. 1A) [20]–[22], [27], direct confirmation of the *Fv2* genotype of 129P2 mice would be ideal. Recently, infection of 129P2 mice with an N-tropic MuLV strain, CasFrKP, resulted in high infection levels [28]. These results contrasted from the lack of FV viremia following B-tropic FV infection of 129P2 mice [7]. Thus, the use of a B-tropic [7] versus an N-tropic [28] MuLV strain resulted in divergent infection outcomes in 129P2 mice.

*Fv1* is the gene that dictates N- versus B-tropism [29]. Mouse strains classified as *Fv1* ^n/n^ support the replication of N-tropic FV strains, whereas *Fv1* ^b/b^ mice support B-tropic FV replication [30], [31]. Some FV strains can infect both *Fv1* ^n/n^ and *Fv1* ^b/b^ mouse strains efficiently and are considered as dual- or NB-tropic. *Fv1* incompatibility, such as N-tropic FV infection of *Fv1* ^b/b^ mice, results in a potent post-entry block [30], [31] that map to amino acid differences in *Fv1* and the F-MuLV capsid [32]–[35]. Interestingly, another 129 substrain, 129S8 (formerly 129/SvEv [20]) was genotyped as *Fv1* ^nr^, a variant of *Fv1* ^n^ [33], [36]. Fv1^nr^ restricts B-tropic as well as some N-tropic strains. The *Fv1* data on 129S8 [33], [36] strongly suggest that 129P2 mice should also be *Fv1* ^nr/nr^. However, the 129 sublines 129/Re and 129/Rr that gave rise to 129P2 and 129S8, respectively, were separated and independently maintained since 1948 (Fig. 1A). Thus, confirmation that 129P2 mice are *Fv1* ^nr/nr^ may be warranted. We therefore re-evaluated the *Fv1*, *Fv2* and *Rfv3* genotype status of 129P2 mice and confirmed these genotypes with *in vivo* infection with a dual-tropic FV strain. The results demonstrate that the previous classification of 129P2 mice as *Fv2* and *Rfv3* resistant based on phenotypic data [7] was confounded by *Fv1* incompatibility. Importantly, this endeavor revealed new insights on the virological and immunological impact of *Apobec3* and *Fv1* restriction in 129P2 mice, a mouse genetic background that may be useful for basic retroviral vaccine studies.

## Materials and Methods

### Mice

Wild-type (WT) 129P2 mice were purchased from Harlan Laboratories, Incorporated. *Apobec3* knock-out (KO) mice were generated from the XN150 embryonic stem cell line (BayGenomics) that was made in the 129P2 background. The chimeric mouse was backcrossed once to 129P2 to generate a 100% congenic 129P2 *Apobec3* KO strain [7]. B6, A.BY and BALB/c mice were obtained from The Jackson Laboratory. Experimental protocols in mice specifically for this study were approved by the Institutional Animal Care and Use Committee at the University of Colorado Denver [Permit Number B-89709(10)1E]. Infections were performed under isoflurane anesthesia. At indicated timepoints, mice were euthanized using a double-procedure consisting of carbon dioxide inhalation followed by cervical dislocation. All efforts were made to minimize suffering.

### Cell culture

*Mus dunni* and 293T cells were maintained in DMEM (Mediatech) with 2% penicillin/streptomycin/glutamine (Mediatech) and 10% fetal bovine serum (FBS; Gemini).

### Genotyping

Tail DNA from 129P2, B6 (*Fv1^b/b^ Fv2^r/r^ Rfv3^r/r^*) and BALB/c (*Fv1^b/b^ Fv2^s/s^ Rfv3^s/s^*) mice were extracted using the DNAEasy Kit (Qiagen). To genotype *Fv1*, the full-length gene was amplified using a forward primer Fv1.F (5′-AAGCTTGCGGCCGCGAATTTCCCACGTGCGCTTGCT) and a reverse primer that was either *Fv1* ^b^-specific, Fv1b.R (5′-GAATCCTCTAGACTATTAACTGTTGCTTTGATGTTTC), or *Fv1* ^n^-specific (5′GAATCCTCTAGACTATCAGAGTTTTGTAGCTGCTGT). PCR products were directly purified (Qiagen), sequenced, and compared to published *Fv1* alleles.

To genotype *Fv2*, we designed primers flanking a 3 nt (GGA) indel in the *Stk* intron 10 of *Fv2* susceptible strains. This indel results in an alternative promoter that drives the transcription of a short-form of the Stk kinase (sf-Stk) [24]. The primers were Stk.Fv2.F (5′-CAGTCCCCTGATGTCCAACT) and Stk.Fv2.R (5′-CCACGGTCATGTTCACAGTC). DNA (100 ng) was subjected to PCR amplification in a 50 μl reaction that contained 1× Phusion HF buffer (Thermo Scientific), 0.2 μM primers, 20 μM dNTP (New England Biolabs), and 1 U of Phusion High-fidelity DNA polymerase (New England Biolabs). The cycling conditions consisted of a 98°C initial denaturation step for 30 s followed by 36 cycles of 98°C for 10 s, 59°C for 15 s, and 72°C for 15 s, and a final extension step of 72°C for 7 min. The 450-bp PCR products were purified (Qiagen) and directly sequenced.

To genotype *Rfv3*, a primer pair flanking the X-MLV LTR insertion in the exon 2 splice site of *Apobec3* was used for PCR [12], [16]. PCR amplicons were sized by agarose gel electrophoresis.

### Quantification of *Apobec3* mRNA levels

Spleen RNA was extracted using the RNAEasy kit and *Apobec3* mRNA levels were quantified by real-time PCR as described [12] and normalized against GAPDH. *Apobec3* mRNA levels from spleens of 2 mice of each strain were quantified in triplicate. Quantitative real-time PCR was performed in a Biorad CFX6 machine.

### Virus stocks and FV infection

B-tropic and NB-tropic FV stocks, previously obtained from Kim Hasenkrug, Leonard Evans and Bruce Chesebro at the Rocky Mountain Laboratories, National Institutes of Allergy and Infectious Diseases, were prepared and titered in BALB/c mice [7], [37]. Both stocks contain F-MuLV, SFFV and lactate-dehydrogenase elevating virus (LDV). LDV is an RNA virus endemic in wild mice [38] and was present in the classical FV stocks used to define *Fv1, Fv2, H-2* and *Rfv3* [39]. Since this study builds on historical FV studies that defined *Fv1, Fv2, H-2* and *Rfv3*, FV/LDV stocks were required. Infections were performed by intravenous injection through the retro-orbital route of 140 to 7,500 Spleen Focus Forming Units (SFFU) of FV. Mock infections were performed using DMEM without FBS. Mice were sacrificed at indicated timepoints. In some experiments, 129P2 *Apobec3* KO mice that were previously infected with B-tropic FV were challenged with an NB-tropic strain at 28 dpi.

### Infectious viremia

Infectious viremia was determined using a focal infectivity assay involving incubations of infected *Mus dunni* cells with FV gp70-specific MAb 720 [7], [40]. Briefly, serial dilutions of plasma were added into *Mus dunni* cells seeded the previous day with polybrene, fixed after 2–3 days with ethanol, then MAb 720 supernatant was added for 3 h at 37°C. The fixed cells were washed 3× with TNE buffer (10 mM Tris, 200 mM NaCl, 1 mM EDTA, pH = 7.4) with 0.25% Tween-20 (TBS-T), then 1∶500 sheep anti-mouse IgG conjugated to horseradish peroxidase (HRP; Amersham) was added for 1 h. The cells were washed 3× with TNE then developed with aminoethylcarbazole (Sigma) substrate in the presence of H_2_O_2_ (Sigma). Foci were counted and expressed as focal infectivity units (FFU) per ml of plasma.

### Spleen infectious center assay

A day before harvesting spleens, *Mus dunni* cells were seeded at 8,000 cells per well in 24-well plates and 32,000 cells per well in 6-well plates in media with polybrene. The next day, spleens were pre-weighed, and no more than 400 mg were disaggregated and diluted to 10 ml with complete media. For the 6-well plates, 1 ml of spleen suspension was added, while for 24-well plates, the splenocytes were serially diluted 10-fold, adding 100 µl of cell suspension. Infectious centers were detected using the same procedure for the infectious viremia assay described above, using MAb 720 supernatant as the detecting antibody. Foci were counted, multiplied by the dilution factor for the entire spleen, and expressed as spleen infectious centers per spleen.

### Plasma viral load

Viral RNA copy numbers were quantified from 10 µl plasma by quantitative real-time PCR [12], [41]. T7-transcribed RNA was used as a standard for absolute quantification. The assay has a limit of detection of 10^3^ copies/ml and >95% efficiency.

### Endpoint FV-specific IgG titer

Endpoint ELISAs were performed as described [41]. Native FV virions were coated (500 ng/well) into Immulon-4 plates overnight at 4°C then blocked for 2 h with Superblock (Pierce). Serial 2-fold dilutions of plasma were added onto the plate, incubated at 37°C for 1 h, then washed 6× with PBS with 0.25% Tween-20 (PBS-T). Biotinylated goat anti-mouse IgG (Southern Biotechnology) was added at 1:4000 and incubated at 37°C for 1 h then washed 6× with PBS-T. Streptavidin conjugated to HRP was added at 1:4000 and incubated at 37°C for 30 min then washed 6× with PBS-T, followed by the addition of 3,3′,5,5′-tetramethylbenzidine (TMB) substrate (BioFX). Reactions were stopped with 0.3N H_2_SO_4_, then read in a Victor X5 (Perkin Elmer) plate reader at 405 nm. Endpoint titers were calculated by interpolating 2× the average background per plate from a best-fit nonlinear regression curve calculated using Prism 5.0 software (GraphPad).

### NAb assay

NAb titers were determined by incubating serial dilutions of plasma with 150 FFU of N-tropic F-MuLV for 1 h at 37°C and adding a third of the mixture onto plated *Mus dunni* cells. FV infection foci were counted [7], and 80% inhibitory concentrations were calculated by interpolation from a best-fit nonlinear regression curve using Prism 5.0 (GraphPad).

### Flow cytometry

To assess cell-mediated immune responses, splenocytes (10^6^ cells) were stimulated for 5 h with PMA (25 ng/ml) and ionomycin (0.7 ng/ml) (Sigma) at 37°C and simultaneously stained with anti-CD107a-PE-Cy7 (clone ID4B) (BD Biosciences). Brefeldin A (100 µg/ml) (Sigma) was added after 1 h. Cells were washed 2× in FACS buffer (PBS + 1% FBS), stained with anti-CD4-PE-CF594 (RM4-5), CD8-FITC (53-6.7) and CD49b-APC (DX5) (BD Biosciences) for 30 min at 4°C, washed 2×, then permeabilized and fixed in Perm/fix buffer (BD Biosciences) prior to staining with anti-IFNγ-PE (XMG1.2) (BD Biosciences). To assess FV infection levels, BM and splenocytes were stained for 1 h at 4°C with MAb34, a monoclonal antibody against the FV Glyco-Gag protein, as previously described [42]. The cells were washed once in FACS buffer, then costained with goat anti-mouse IgG2b-APC (Columbia Biosciences). Cells were also stained with antibodies to CD3-AlexaFluor700 (17A2), CD19-APC-H7 (1D3), CD11b-PE-CF594 (MI/70) (BD Biosciences); Ter119-FITC (TER-119) and CD11c-PE-Cy7 (N418) (eBioscience). To monitor B cell subpopulations, cells were stained with anti-B220-PerCP (RA3-6B2), CD138-PE (281-2), GL7-FITC (GL7) (BD Biosciences) and IgD-eFlour450 (11-26c) (eBioscience) for 30 min at 4°C. For all stainings, the cells were washed then fixed in 1% paraformaldehyde in PBS. Isotype controls were used for gate construction. Samples were analyzed on an LSR II (BD Biosciences) flow cytometer, collecting 200,000–500,000 events per sample. Data were analyzed using FlowJo (Tree Star).

### Statistical analysis

Datasets with a normal distribution based on the Kolmogorov-Smirnov normality test were analyzed using a 2-tailed Student’s *t* test. Otherwise, a 2-tailed Mann-Whitney U test was used. Statistical analyses were performed using the Prism 5.0c (GraphPad). *P* values less than 0.05 were considered statistically significant.

## Results

### *Fv1, Fv2* and *Rfv3* genotypes of 129P2 mice

The 129 mouse lineage exhibits a complex genealogy resulting in multiple substrains (Fig. 1A). Thus, the *Fv1, Fv2* and *Rfv3* genotypes of 129P2 mice, a representative of the ‘Parental’ 129 substrains, were re-evaluated. As expected, sequencing of the entire *Fv1* gene of 129P2 mice revealed 100% nucleotide and amino acid identity to the previously reported *Fv1^nr^* sequence from 129S8 [36]. The C-terminal residues were distinct from Fv1^b^ as previously reported. In contrast to Fv1^n^ and Fv1^b^ which encode a Ser at position 352, the 129P2 Fv1 encoded a Phe, consistent with its designation as Fv1^nr^ [36]. To genotype *Fv2*, a segment of the *Stk* gene encompassing a 3-nt indel (GGA) that dictates alternative transcription of sf-Stk [24] was amplified from DNA of 129P2, B6 (*Fv2* ^r/r^), BALB (*Fv2* ^s/s^) and A.BY (*Fv2* ^s/s^) strains. 129P2 mice encoded the GGA insertion, similar to BALB and A.BY, but in contrast to B6. To genotype *Rfv3*, a primer set encompassing a 530 bp X-MLV LTR insertion in the *Apobec3* exon 2 splice site [12], [16] was used for PCR. These analyses revealed that 129P2 mice lacked this X-MLV LTR insertion in *Apobec3* (Fig. 1B), similar to *Rfv3* ^s/s^ BALB and A.BY mice. In addition, 129P2 mice had >10-fold lower *Apobec3* mRNA levels in the spleen compared to B6 mice (Fig. 1C). Thus, based on genotyping, 129P2 mice are *Fv1* ^nr/nr^ *Fv2* ^s/s^ *Rfv3* ^s/s^ (Table 1).

### Infection of 129P2 WT and *Apobec3* KO mice with B-tropic versus NB-tropic FV

**Figure 2:**
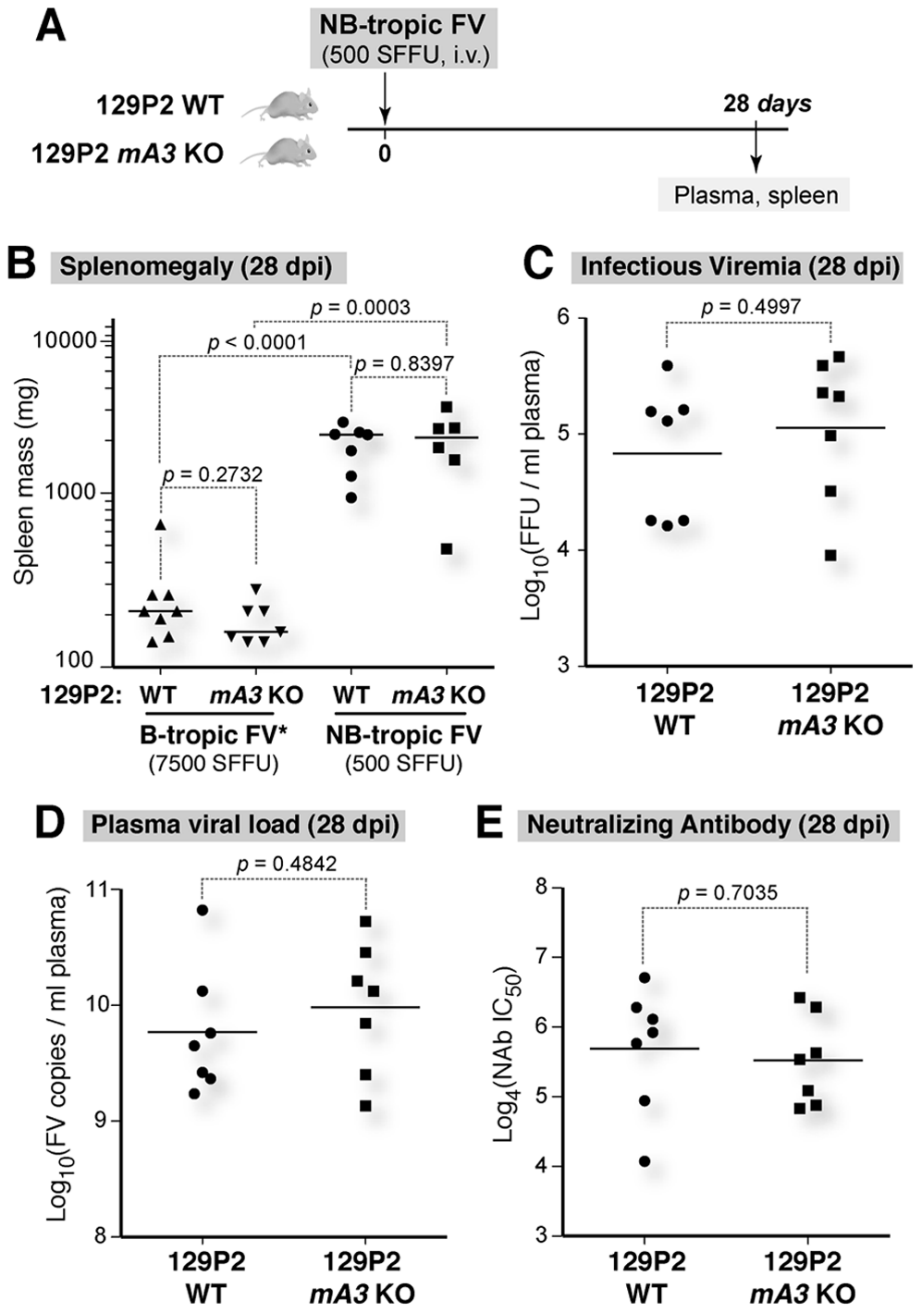
129P2 *Apobec3* does not promote recovery from viremia and disease following NB-tropic FV infection. (A) Infection schedule. Mice were sacrificed at 28 dpi for subsequent analyses. (B) Spleen mass. A normal mouse spleen would have ∼100 mg of wet spleen weight. NB-tropic FV infection induced severe splenomegaly but not B-tropic FV. (C) Plasma infectious viremia, based on a focal infectivity assay in *Mus dunni* cells. (D) Plasma viral load, based on quantitative PCR. (E) NAb titer, calculated based on neutralization curves following incubation of serial dilutions of heat-inactivated plasma with FV in *Mus dunni* cells. For panels B to E, black bars represent the mean, and each dot corresponds to an individual mouse. Statistical analyses were performed using a 2-tailed Student’s *t*-test, and *p* values <0.05 were considered statistically significant.

High-dose (7500 SFFU) infection of 129P2 mice with B-tropic FV did not induce splenomegaly and resulted in potent NAb responses, prompting the earlier designation of 129P2 mice as *Fv2* ^r/r^ and *Rfv3* ^r/r^ [7]. However, these results were likely due to *Fv1* incompatibility, since 129P2 mice are *Fv1* ^nr/nr^ (Table 1). To overcome a potential *Fv1* restriction block, 129P2 mice were infected with NB-tropic FV, which should infect mice regardless of *Fv1* genotype [37]. The mice were sacrificed at 28 dpi (Fig. 2A), because recovery from viremia and NAb responses could be reproducibly detected by this timepoint [9], [10].

Consistent with previous data [7], high-dose B-tropic FV infection did not elicit severe splenomegaly in 129P2 mice (median spleen mass  =  0.21 g) at 28 dpi. In sharp contrast, NB-tropic FV infection resulted in 10-fold higher splenomegaly (median spleen mass  =  2.2 g), despite a 15-fold lower inoculum dose compared to B-tropic FV infection (Fig. 2B). This result confirms that 129P2 mice are *Fv2* ^s/s^.

In a previous study, CasFrKP MuLV that lacks the putative Apobec3 antagonist, Glyco-Gag, replicated to higher levels in 129P2 *Apobec3* KO versus WT mice [28]. These results, coupled with data showing that 129P2 Apobec3 can significantly inhibit F-MuLV *in vitro* [7], suggested that 129P2 *Apobec3* is a functional antiretroviral restriction factor. However, the virological and immunological impact of 129P2 *Apobec3* in pathogenic FV infection remains unknown. We therefore infected 129P2 WT and *Apobec3* KO mice with NB-tropic FV and evaluated infection parameters at 28 dpi. There was no significant difference in splenomegaly between 129P2 WT and *Apobec3* KO mice (Fig. 2B). Importantly, plasma viral load (Fig. 2C), infectious viremia (Fig. 2D) and NAb titer (Fig. 2E) at 28 dpi were not significantly different between 129P2 WT and *Apobec3* KO mice. Thus, the 129P2 *Apobec3* allele did not promote recovery from viremia and NAb responses during pathogenic FV infection, consistent with the genotype status of 129P2 mice as *Rfv3* ^s/s^.

### B-tropic FV infection of *Apobec3*-deficient 129P2 mice induced significant cell-mediated and humoral immune responses

The finding that B-tropic FV did not induce splenomegaly in *Fv1* ^nr/nr^ 129P2 mice suggested that FV replication was blocked due to *Fv1* incompatibility. This observation mirrored previous results with N-tropic FV infection of *Fv1* ^b^ mice, where N-tropic FV replicated at near-undetectable levels [43]–[47]. Interestingly, N-tropic FV infection of *Fv1* ^b^ mice resulted in substantial protection from pathogenic B-tropic FV challenge [43]–[46] (Table 2). N-tropic FV induced potent cellular and humoral immune responses that can be adoptively transferred into naïve hosts [45], [47]. Thus, N-tropic FV functioned as a live-attenuated vaccine in an *Fv1*-incompatible host. However, the reverse scenario with B-tropic FV had not previously been tested.

**Table 2:** Retrovirus vaccine studies based on *Fv1* incompatibility.

		Genotype							
Year	Host strain	*Fv1*	*Fv2*	*H2* a	*Rfv3*	Vaccine strain^b^	Challenge Strain^b^	Results	Ref.
1986	(B10.A × A.BY)F_1_	*b/b*	*r/s*	*a/b*	*r/s*	N-tropic FV complex	B-tropic FV complex	Protection from challenge independent of *H2*	[43]
	(B10.A × A/WySn)F_1_	*b/b*	*r/s*	*a/a*	*r/s*	N-tropic FV complex	B-tropic FV complex	Protection from challenge independent of *H2*	[43]
1998	(B10.A × A/WySn)F_1_	*b/b*	*r/s*	*a/a*	*r/s*	N-tropic F-MuLV helper^c^	B-tropic FV complex	Protection due to immunity, not receptor interference	[44]
1999	(B10.A × A.BY)F_1_	*b/b*	*r/s*	*a/b*	*r/s*	N-tropic F-MuLV helper^c^	B-tropic FV complex	T-cell protection transferrable to naïve host	[45], [46]
2004	B6 (adoptive transfer)	*b/b*	*r/r*	*b/b*	*r/r*	N-tropic F-MuLV helper^c^	B-tropic FV complex	Virus-specific antibodies are critical for protection	[47]
2013	129P2 *Apobec3* KO	*nr/nr*	*s/s*	*b/b*	*null*	B-tropic FV complex	NB-tropic FV complex	Protection from challenge independent of *Apobec3*	This study

a *H2* dictates cell-mediated immune responses, with the *b* haplotype being more protective than *a*. ^b^Entries designated as FV complex correspond to the classical FV stocks that contain F-MuLV helper virus, SFFV and LDV. ^c^Without SFFV, F-MuLV is nonpathogenic and does not cause splenomegaly.

**Figure 3:**
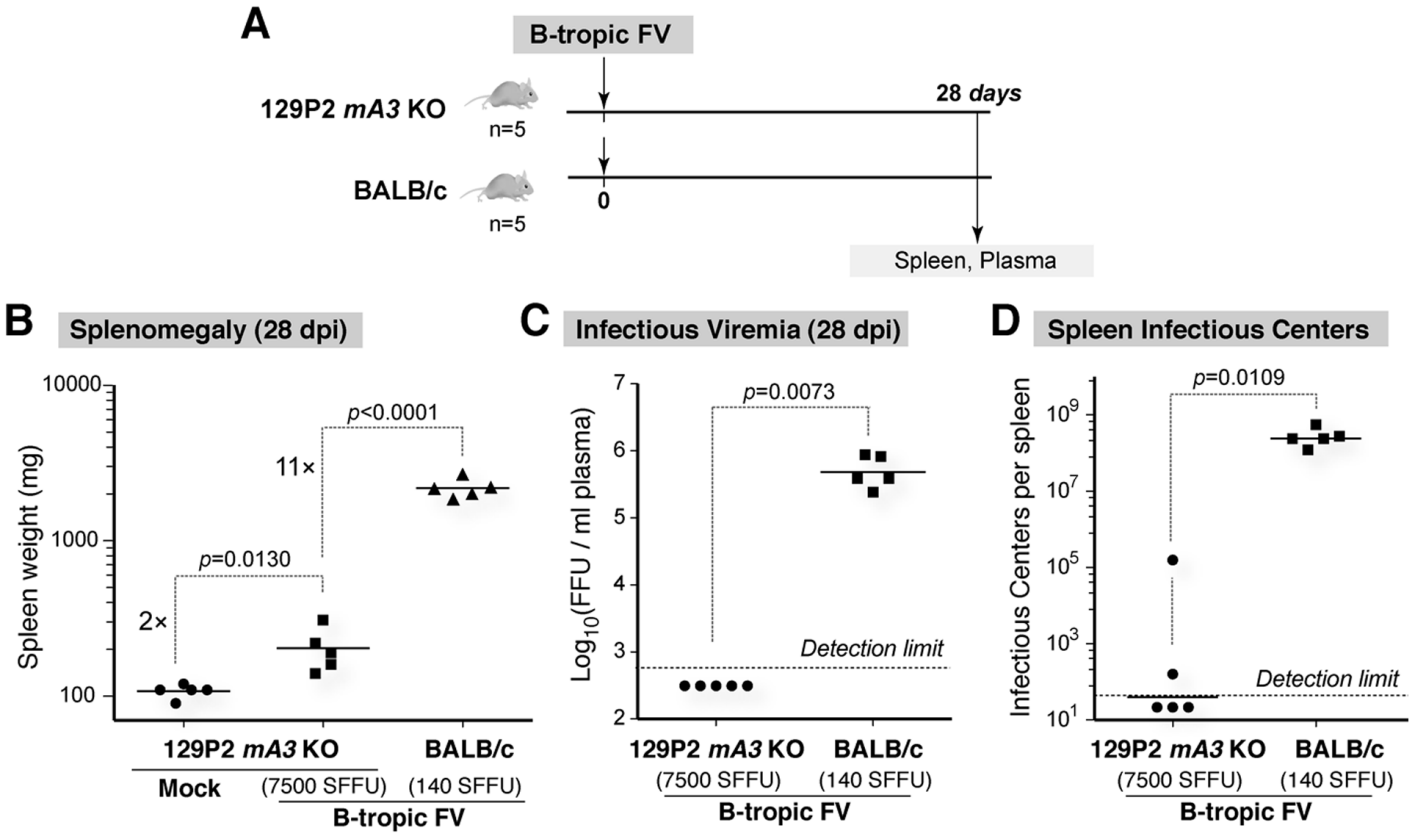
B-tropic FV replication in 129P2 *Apobec3* KO versus BALB/c mice. (A) Infection schedule. At 28 dpi, mice were sacrificed for analyses. (B) Spleen mass. Severe splenomegaly was observed in BALB/c, but not 129P2 *Apobec3* KO mice following B-tropic FV infection. Data were analyzed using a 2-tailed Student’s *t* test. (C) Infectious viremia. B-tropic FV infection of 129P2 *Apobec3* KO mice resulted in undetectable infectious viremia using the focal infectivity assay in *Mus dunni* cells. The limit of detection is 600 FFU/ml plasma. (D) Spleen infectious centers. Titrated amounts of 28 dpi splenocytes were co-incubated with *Mus dunni* cells and developed using the focal infectivity assay procedure. The data in panels C and D were analyzed using a 2-tailed Mann-Whitney U test. For panels B to D, *p* values <0.05 were considered statistically significant. Black bars represent the mean, and each dot corresponds to an individual mouse. Data are representative of two independent experiments.

We therefore investigated if B-tropic FV can function as a live-attenuated vaccine in 129P2 mice. We utilized 129P2 *Apobec3* KO mice to ensure that *Apobec3* will have no impact on the results. Although the 129P2 *Apobec3* allele did not influence NAb responses and recovery from pathogenic FV infection (Fig. 2), 129P2 Apobec3 may still restrict less pathogenic or attenuated MuLV strains [28]. 129P2 *Apobec3* KO mice were infected with either mock (DMEM) or high-dose (7500 SFFU) B-tropic FV. At 28 dpi, the mice were evaluated for FV infection and immune responses (Fig. 3A). As expected, B-tropic FV infection of 129P2 *Apobec3* KO mice did not induce severe splenomegaly (Fig. 3B) and infectious titers were below the limit of detection (≤600 FFU/ml) in the plasma (Fig. 3C). In contrast, infection of BALB/c mice with 53-fold lower dose (140 SFFU) of the same B-tropic FV stock induced 11-fold higher splenomegaly (Fig. 3B). Moreover, assuming that the infectious titer in B-tropic FV infected 129P2 mice is the limit of detection (600 FFU/ml), the same virus stock induced at least an 800-fold higher infectious viremia in BALB/c mice (Fig. 3C). The relatively mild splenomegaly induced by B-tropic FV infection of 129P2 *Apobec3* KO mice (Fig. 3B) suggested that B-tropic FV replicated below the detection limit of the plasma infectious viremia assay (Fig. 3C). We therefore measured FV infection levels using the more sensitive spleen infectious center assay, which involves co-incubating splenocytes with *Mus dunni* cells. As expected, this assay revealed very high (>10^8^) spleen infectious centers in BALB/c mice, whereas only 2 of 5 129P2 *Apobec3* KO mice had detectable signals (Fig. 3D). We also evaluated 129P2 wild-type mice infected with B-tropic FV at 28 dpi and found only 2 of 4 infected mice had detectable spleen infectious centers (data not shown). The mild splenomegaly, undetectable infectious viremia and sporadic detection of spleen infectious centers suggested that B-tropic FV replicated at very low levels in 129P2 *Apobec3* mice by 28 dpi, and was therefore ‘live-attenuated’.

**Figure 4:**
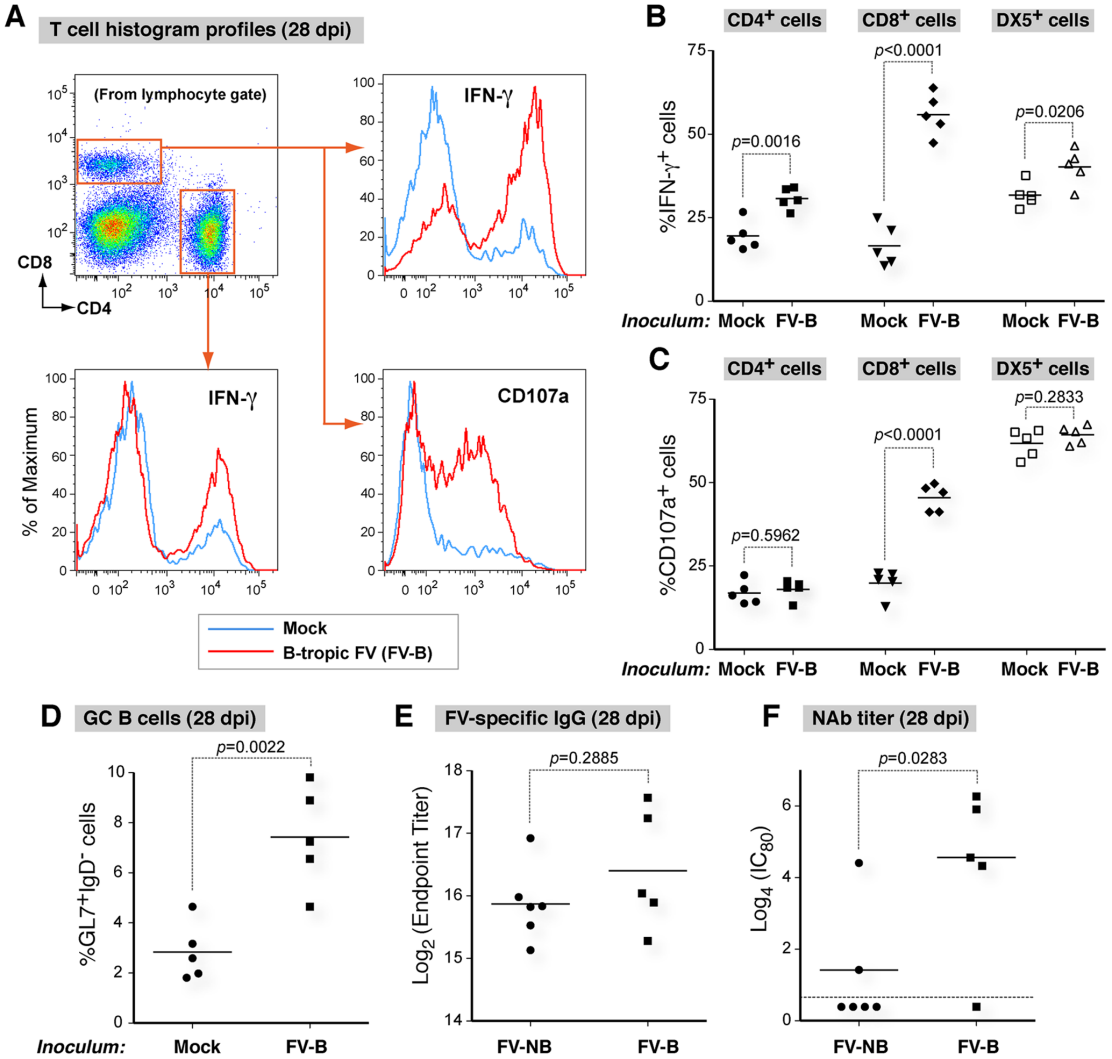
*Fv1* incompatibility induces significant cell-mediated and humoral immune responses. (A–D) *Apobec3*-deficient 129P2 mice were infected with 7500 SFFU B-tropic FV (FV-B) or DMEM (mock). Mouse splenocytes were analyzed by flow cytometry at 28 dpi. (A–C) Cell mediated immune responses. Splenocytes were stimulated with PMA/ionomycin for 5 h prior to flow cytometric analysis. (A) Representative pseudo-color plots and histograms. Flow cytometry plots show the gating strategy for CD4+ and CD8+ lymphocytes. (B) Intracellular IFN-γ and (C) Surface CD107a in lymphocyte subsets. (D) Splenic germinal center (GL7+ IgD-) B cells. (E-F) Analysis of FV-specific antibody responses in 28 dpi plasma of 129P2 *Apobec3* KO mice following infection with B-tropic FV (7500 SFFU) or NB-tropic FV (500 SFFU). (E) FV-specific IgG endpoint titers as measured by ELISA. (F) NAb titers. For panels B to F, the black bars represent the mean, and each dot corresponds to an individual mouse. Data were analyzed using a 2-tailed Student’s *t* test with *p*<0.05 considered as significant. Data are representative of two independent experiments.

We next evaluated the induction of immune responses by flow cytometry (Fig. 4A). Compared to uninfected controls, significant induction of IFN-γ in stimulated CD4+ T cells, CD8+ T cells and DX5+ NK cells were observed (Fig. 4B). Significant induction of CD107a, a degranulation marker, was observed in CD8+ T cells but not in CD4+ T cells and DX5+ cells (Fig. 4C). In addition, splenic germinal center (GL7+ IgD-) B cells were significantly induced in mice infected with B-tropic FV (Fig. 4D). Using 28 dpi plasma from 129P2 *Apobec3* KO mice infected with pathogenic NB-tropic FV as a reference, we observed similar FV-specific endpoint IgG titers from 28 dpi plasma of B-tropic FV infected 129P2 *Apobec3* KO mice (Fig. 4E). However, significantly higher NAb responses (Fig. 4F) were observed in B-tropic FV infected 129P2 *Apobec3* KO mice. These findings demonstrate that B-tropic FV infection of 129P2 *Apobec3* KO mice induced significant cell-mediated and humoral immune responses.

### B-tropic FV infection of *Apobec3*-deficient 129P2 mice protects from pathogenic NB-tropic FV challenge

**Figure 5:**
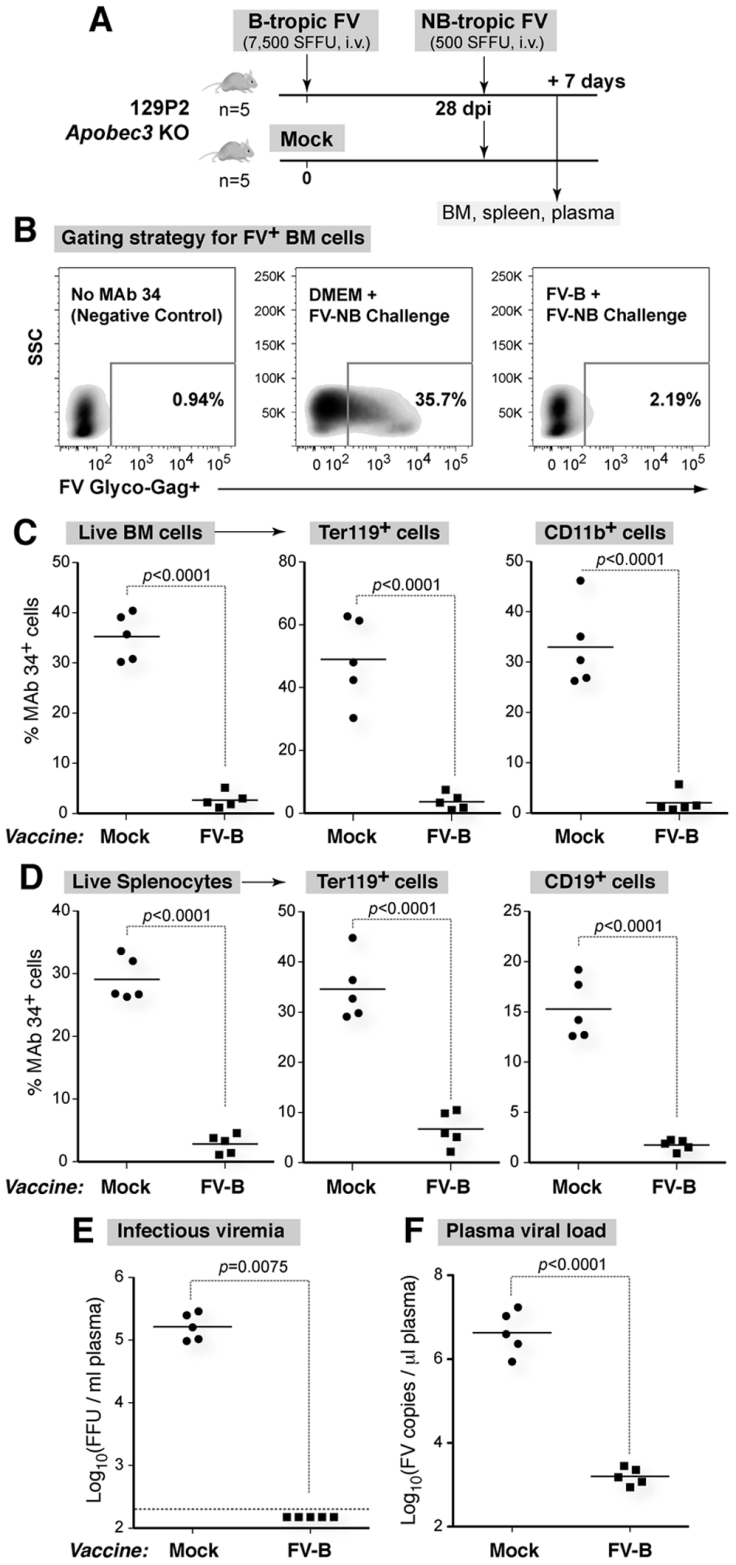
B-tropic FV protected 129P2 *Apobec3* KO mice from pathogenic NB-tropic FV challenge. (A) Infection schedule. 129P2 *Apobec3* KO mice were infected with B-tropic FV (FV-B) or mock (DMEM). At 28 dpi, the mice were challenged with NB-tropic FV (FV-NB) and evaluated for infection levels by flow cytometry 7 days later. (B) Gating strategy for FV+ cells. BM cells and splenocytes (not shown) were incubated with an FV Glyco-Gag-specific IgG2b antibody, MAb 34, then stained with a goat anti-mouse IgG2b antibody conjugated to APC. Representative flow plots demonstrating the gating strategy for MAb 34+ cells are shown. Cellular infection levels at 7 days post-challenge are shown for (C) BM and (D) Splenocytes. Ter119+ erythroblasts, CD11b+ myeloid cells and/or CD19+ B cells were gated from the live population. Plasma samples were analyzed for (E) Infectious viremia based on a focal infectivity assay in *Mus dunni* cells and (F) plasma viral load based on viral RNA copies detected by quantitative RT-PCR. Black bars represent the mean, and each dot corresponds to an individual mouse. Flow cytometry and plasma viral load data were analyzed using a 2-tailed Student’s *t* test. Infectious viremia data were analyzed using a 2-tailed Mann-Whitney U test. Data were considered statistically significant with *p*<0.05. Data are representative of two independent experiments.

We next evaluated whether B-tropic FV infection of 129P2 *Apobec3* KO mice (Fig. 5) will protect from subsequent infection with pathogenic NB-tropic FV. Mice previously inoculated with either DMEM or B-tropic FV were challenged with pathogenic NB-tropic FV at 28 dpi (Fig. 5A). FV infection levels were determined 7 days later by flow cytometry (Fig. 5B). Significantly lower cellular FV infection in BM cells as well as major BM target cells that include Ter119+ erythroblasts and CD11b+ myeloid cells were observed in B-tropic FV ‘vaccinated’ mice (Fig. 5C). Significant protection was also observed in the spleen, including erythroblasts and B cells (Fig. 5D). Mice previously inoculated with B-tropic FV had undetectable infectious plasma viremia at 7 days post-challenge with NB-tropic FV, whereas mock-infected mice showed high levels of infectious plasma viremia (Fig. 5E). More sensitive plasma viral load assays showed that mice previously inoculated with B-tropic FV had >2500 fold lower viral RNA load in the plasma compared to mock (Fig. 5F). Thus, B-tropic FV acted as a live-attenuated vaccine in *Fv1-*incompatible 129P2 mice.

## Discussion

The Friend retrovirus (FV) infection model was instrumental in the identification of host genes that can influence the retrovirus-specific immune response. One classical gene, *Rfv3*, influences recovery from FV viremia by modulating the NAb response, and was recently found to be encoded by the innate restriction factor *Apobec3*. However, the correlation between *Apobec3* polymorphisms and *Rfv3* genotype is challenged by uncertainties in the *Apobec3/Rfv3* genotype of an inbred strain known as 129P2. We therefore resolved the FV resistance genotype and phenotype of 129P2 mice. The results revealed that 129P2 are resistant to B-tropic FV (*Fv1* ^nr/nr^), are susceptible to splenomegaly (*Fv2* ^s/s^), and encode an *Rfv3*-susceptible allele of *Apobec3* (Table 1). The latter result provided a unique opportunity to evaluate the *Rfv3*-susceptible allele of *Apobec3*. Most studies on *Apobec3/Rfv3* have focused on the B6 *Apobec3* allele, which exhibit properties such as higher levels of expression that make it more potent *in vivo* [11]-[16]. Here we show that the 129P2 *Apobec3* allele did not promote recovery from viremia and NAb responses relative to *Apobec3-*null mice, consistent with *Rfv3* susceptibility. Thus, the results resolve issues on the concordance between *Apobec3* polymorphisms and *Rfv3* genotype status with respect to the 129P2 strain, and further strengthen the case for *Apobec3* as the gene encoded by *Rfv3.*

The lack of replication of B-tropic FV in *Fv1* ^nr/nr^ 129P2 mice mirrored earlier studies involving N-tropic FV infection of *Fv1* ^b/b^ mice (Table 2). These studies showed that N-tropic FV could function as a live-attenuated vaccine in an *Fv1-*incompatible host, resulting in the best immunological protection so far against FV infection [5]. Thus, *Fv1* incompatibility may be an important mechanism to augment retrovirus-specific adaptive immune responses. However, these prior *Fv1*-restricted vaccine studies utilized mice that encode the B6 *Apobec3* allele and are therefore *Rfv3* resistant (Table 2). In other words, the resulting protective adaptive immune response may not be exclusively due to *Fv1* restriction, as B6 *Apobec3* is known to prime a stronger NAb response [7], [11], [12]. In addition, the reverse scenario involving B-tropic FV ‘vaccination’ of an *Fv1* ^n/n^ host had not yet been performed. Thus, the potential contribution of B6 *Apobec3* in prior *Fv1* vaccine studies, and the lack of data on B-tropic FV as a live-attenuated vaccine, raise uncertainties on whether *Fv1* restriction is a general retrovirus vaccine concept in mice.

We therefore performed a vaccination study to test if B-tropic FV can function as a live-attenuated vaccine. We utilized 129P2 *Apobec3* KO mice to ensure that 129P2 *Apobec3* will not contribute to the vaccine-elicited immune response. Even though the 129P2 *Apobec3* allele did not promote NAb responses and recovery from FV infection, these phenotypes were observed in the context of pathogenic FV infection. *Apobec3* restriction is saturable *in vivo* [41], and by 28 dpi, high replication levels may have overwhelmed a less potent 129P2 *Apobec3* restriction phenotype [28]. Thus, 129P2 *Apobec3* KO mice were ‘vaccinated’ with B-tropic FV and after 28 days, challenged with pathogenic, NB-tropic FV. Our results revealed that B-tropic FV replicated at near-undetectable levels by 28 dpi, induced significant cell-mediated and humoral immune responses and protected mice from pathogenic NB-tropic FV infection independent of *Apobec3*. Thus, *Fv1* restriction was sufficient to induce immunological protection, strengthening the case for *Fv1* incompatibility as a retrovirus vaccine paradigm in mice. Since *Fv1* restriction prevented splenomegaly and promoted NAb responses in *Fv2* and *Rfv3* susceptible mice, the data also demonstrate that in 129P2 mice, *Fv1* restriction is dominant over *Fv2* resistance and *Apobec3* restriction. This finding is consistent with a proposed ‘restriction factor hierarchy’ [37], whereby dominant resistance mechanisms may mask the impact of other restriction mechanisms *in vivo*.

The mechanism for how *Fv1* incompatibility elicited potent adaptive immunity could readily be explained by live-attenuation: FV replicated at low levels, thereby inducing a protective immune response [43]–[47]. However, the quality of the immunological response induced by *Fv1* incompatibility raise the question of whether signaling pathways that potentiate and/or amplify the adaptive immune response were also induced. Recently, HIV-1 capsid recognition by TRIM5α induced downstream AP-1 and NF-κB regulated genes that may orchestrate the adaptive immune response [48]. TRIM5α exhibited all the attributes of a pattern-recognition protein or innate sensor [48]. Nanotube reconstitution studies revealed that Fv1 recognizes MLV capsids in a lattice arrangement [49], analogous to TRIM5α. Thus, we speculate that *Fv1* incompatibility may also trigger a similar cascade of immunity genes. A vaccine model involving B-tropic FV infection of 129P2 mice could prove useful in testing the importance of specific immunological pathways induced by capsid-dependent restriction *in vivo.* 129P2 mice offer advantages that include disease susceptibility that allows for straightforward pathogenic retrovirus challenge studies, and a genetic background that is extensively used in KO/transgenic technologies. It is conceivable that these *Fv1* restriction studies in 129P2 mice may help direct approaches for harnessing TRIM5α biology for human retroviral vaccines.
